# Penta­carbonyl-2κ^5^ *C*-chlorido-1κ*Cl*-bis­[1(η^5^)-cyclo­penta­dien­yl](μ-α-oxido­benzyl­idene-1:2κ^2^ *O*:*C*)titanium(IV)tungsten(0)

**DOI:** 10.1107/S1600536808036465

**Published:** 2008-11-13

**Authors:** Catharine Esterhuysen, I. B. Jacques Nel, Matthias W. Esterhuysen, Stephanie Cronje

**Affiliations:** a Department of Chemistry and Polymer Science, University of Stellenbosch, Private Bag X1, Matieland 7602, South Africa

## Abstract

The title compound, [TiW(C_5_H_5_)_2_(C_7_H_5_O)Cl(CO)_5_], consists of two metal centres, with a (tungstenpenta­carbon­yl)oxy­phenyl­carbene unit coordinated by a titanocene chloride. The oxycarbene group is nearly planar, with the phenyl ring twisted by an angle of 39.1 (2)° with respect to this plane. One of the cyclo­penta­dienyl rings undergoes an offset face-to-face π–π inter­action [3.544 (6) Å] with the symmetry-related cyclo­penta­dienyl ring of a neighbouring mol­ecule.

## Related literature

For related literature regarding anionic Fischer-type carbenes, see: Barluenga & Fañanás (2000 ▶). For information regarding the catalytic activity of similar complexes, see: Luruli *et al.* (2004 ▶, 2006 ▶); Sinn *et al.* (1980 ▶). For comparable structures, see: Esterhuysen *et al.* (2008 ▶); Balzer *et al.* (1992 ▶). For related literature, see: Orpen *et al.* (1989 ▶).
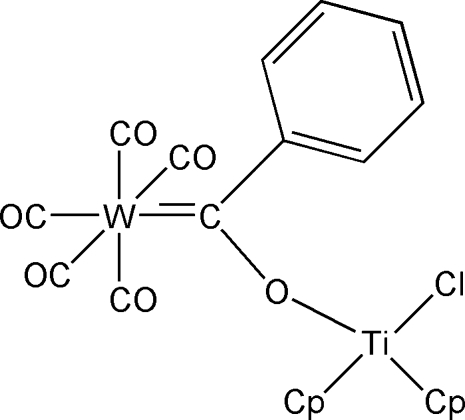


## Experimental

### 

#### Crystal data

[TiW(C_5_H_5_)_2_(C_7_H_5_O)Cl(CO)_5_]
*M* *_r_* = 642.54
Monoclinic, 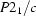

*a* = 8.553 (1) Å
*b* = 12.268 (1) Å
*c* = 20.789 (3) Å
β = 95.903 (1)°
*V* = 2169.8 (3) Å^3^
*Z* = 4
Mo *K*α radiation
μ = 5.83 mm^−1^
*T* = 173 (2) K
0.17 × 0.14 × 0.12 mm

#### Data collection

Nonius KappaCCD diffractometer
Absorption correction: multi-scan (*DENZO-SMN*; Otwinowski & Minor, 1997 ▶) *T*_min_ = 0.438, *T*_max_ = 0.542 (expected range = 0.402–0.497)
12664 measured reflections
4270 independent reflections
3701 reflections with *I* > 2σ(*I*)
*R*_int_ = 0.048

#### Refinement

*R*[*F* ^2^ > 2σ(*F* ^2^)] = 0.025
*wR*(*F* ^2^) = 0.062
*S* = 1.04
4270 reflections
280 parameters
H-atom parameters constrained
Δρ_max_ = 1.05 e Å^−3^
Δρ_min_ = −1.28 e Å^−3^

### 

Data collection: *COLLECT* (Nonius, 1998 ▶); cell refinement: *DENZO-SMN* (Otwinowski & Minor, 1997 ▶); data reduction: *DENZO-SMN*; program(s) used to solve structure: *SHELXS97* (Sheldrick, 2008 ▶); program(s) used to refine structure: *SHELXL97* (Sheldrick, 2008 ▶); molecular graphics: *X-SEED* (Barbour, 2001 ▶; Atwood & Barbour, 2003 ▶); software used to prepare material for publication: *publCIF* (Westrip, 2008 ▶).

## Supplementary Material

Crystal structure: contains datablocks I, global. DOI: 10.1107/S1600536808036465/at2672sup1.cif

Structure factors: contains datablocks I. DOI: 10.1107/S1600536808036465/at2672Isup2.hkl

Additional supplementary materials:  crystallographic information; 3D view; checkCIF report

## Figures and Tables

**Table:** 

W—C1	2.204 (4)
Ti—O1	1.927 (2)
O1—C1	1.280 (4)

**Table:** 

C1—O1—Ti	171.7 (2)

**Fig. 1.**
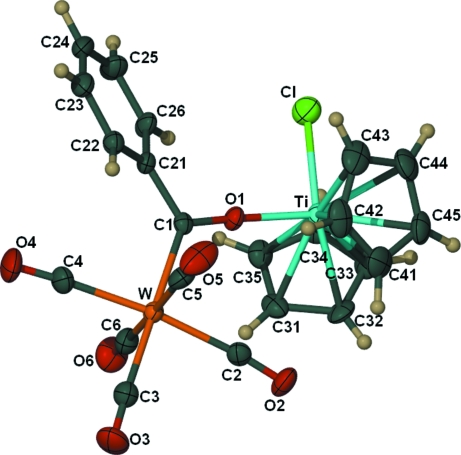
The molecular structure of (I) showing the atomic labelling scheme and displacement ellipsoids drawn at the 50% probability level.

## supplementary crystallographic information

### Comment

Anionic Fischer-type carbene ligands are known to act as monodentate ligands towards transition metals like Ti and Zr (Barluenga and Fañanás, 2000). We have shown that such zirconocene complexes, Cp_2_Zr(Cl)OC(*R*)W(CO)_5_, catalyze the oligomerization of 1-pentene, as well as the copolymerization of ethene and 1-pentene, in the presence of MAO (Luruli *et al.*, 2004; Luruli *et al.*, 2006). Since Cp_2_TiCl_2_ has been shown to polymerize ethylene when activated by methylaluminoxane, MAO (Sinn *et al.*, 1980), the title complex (I) was synthesized as part of our investigation into improved Ziegler-Natta catalysts for polymerization of ethene.

In the title compound (Fig. 1), the W=C_carbene_ and C_carbene_—C distances are similar to those found in the equivalent hafnocene complex [2.177 (6) and 1.291 (6) Å, respectively; Esterhuysen *et al.*, 2008], while the Ti—O distance is similar to the related compound Cp_2_Ti(Cl)OC(C_6_H_5_)Mn(CO)_2_(C_5_H_4_CH_3_) (Balzer *et al.*, 1992). The Ti—O—C angle deviates slightly from linearity, which is similar to the related hafnocene complex [171.4 (3)°], but more linear than the manganese complex [160.8 (5)°]. These results are indicative of π delocalization through the Ti—O—C=W unit. As a result, the Cl/Ti/O1/C1/W/C3/O3 moiety is approximately planar, with the phenyl ring (C21/C22/C23/C24/C25/C26) twisted at an angle of 39.1 (2)° with respect to this plane.

The C31/C32/C33/C34/C35 Cp ring [with centroid *Cg*(1)] undergoes offset face-to-face π–π interactions with the symmetry related Cp ring on a neighbouring molecule [*Cg*(1)···*Cg*(1)^i^ = 3.544 (6) Å; Symmetry code: (i) - *x*, 2 - *y*, 1 - *z*)].

### Experimental

A solution of LiCH_3_ (31.0 ml, 1.6*M*, 50.2 mmol) in 50 ml of diethylether was added to a well stirred suspension of W(CO)_6_ (17.80 g, 50.6 mmol) in 100 ml of diethylether. After solvent removal *in vacuo*, dissolution of the residue in 150 ml of cold water and filtration, a solution of Et_4_NCl (8.72 g, 52.6 mmol) in 50 ml of cold water was added to the filtrate. Upon further filtration 1.13 g (2.0 mmol) of the product {[W(CO)_5_C(C_6_H_5_)O][NEt_4_]} was dissolved in 70 ml of dichloromethane and added to a solution of Cp_2_TiCl_2_ (0.51 g, 2.0 mmol) in 40 ml of dichloromethane. After stirring for 30 min at -40°C AgBF_4_ (0.39 g, 2.0 mmol) was added. The red concentrate, stripped of solvent, was purified by chromatography at -20°C on silica with 400 ml of dichloromethane-pentane (2:1) followed by 200 ml of diethyl ether-hexane (2:1) (column 15 × 2 cm). The eluent was dried *in vacuo*, and the residue dissolved in toluene, layered with pentane and kept at -6°C, whereupon brown crystals of the title compound suitable for X-ray diffraction analysis were obtained in 38% yield.

### Refinement

H atoms were positioned geometrically, with C—H = 0.95 Å, and constrained to ride on their parent atoms, with *U*_iso_(H) = 1.2*U*_eq_(C). The maximum and minimum residual electron density peaks were located 1.05 and 0.86 Å, respectively from the W atom.

### Crystal data

**Table:**

[TiW(C_5_H_5_)_2_(C_7_H_5_O)Cl(CO)_5_]	*F*_000_ = 1232
*M**_r_* = 642.54	*D*_x_ = 1.967 Mg m^−^^3^
Monoclinic, *P*2_1_/*c*	Mo *K*α radiation λ = 0.71073 Å
Hall symbol: -P 2ybc	Cell parameters from 3701 reflections
*a* = 8.553 (1) Å	θ = 1.9–26.0º
*b* = 12.268 (1) Å	µ = 5.83 mm^−^^1^
*c* = 20.789 (3) Å	*T* = 173 (2) K
β = 95.903 (1)º	Prism, brown
*V* = 2169.8 (3) Å^3^	0.17 × 0.14 × 0.12 mm
*Z* = 4	

### Data collection

**Table:**

Nonius KappaCCD diffractometer	4270 independent reflections
Radiation source: fine-focus sealed tube	3701 reflections with *I* > 2σ(*I*)
Monochromator: graphite	*R*_int_ = 0.048
*T* = 173(2) K	θ_max_ = 26.0º
φ and ω scans to fill Ewald sphere	θ_min_ = 1.9º
Absorption correction: multi-scan(DENZO-SMN; Otwinowski & Minor, 1997)	*h* = −10→10
*T*_min_ = 0.438, *T*_max_ = 0.542	*k* = −12→15
12664 measured reflections	*l* = −25→25

### Refinement

**Table:**

Refinement on *F*^2^	Secondary atom site location: difference Fourier map
Least-squares matrix: full	Hydrogen site location: inferred from neighbouring sites
*R*[*F*^2^ > 2σ(*F*^2^)] = 0.025	H-atom parameters constrained
*wR*(*F*^2^) = 0.062	*w* = 1/[σ^2^(*F*_o_^2^) + (0.0268*P*)^2^ + 1.3791*P*] where *P* = (*F*_o_^2^ + 2*F*_c_^2^)/3
*S* = 1.04	(Δ/σ)_max_ = 0.001
4270 reflections	Δρ_max_ = 1.05 e Å^−^^3^
280 parameters	Δρ_min_ = −1.28 e Å^−^^3^
Primary atom site location: structure-invariant direct methods	Extinction correction: none

### Special details

**Table:**

Geometry. All e.s.d.'s (except the e.s.d. in the dihedral angle between two l.s. planes) are estimated using the full covariance matrix. The cell e.s.d.'s are taken into account individually in the estimation of e.s.d.'s in distances, angles and torsion angles; correlations between e.s.d.'s in cell parameters are only used when they are defined by crystal symmetry. An approximate (isotropic) treatment of cell e.s.d.'s is used for estimating e.s.d.'s involving l.s. planes.
Refinement. Refinement of *F*^2^ against ALL reflections. The weighted *R*-factor *wR* and goodness of fit *S* are based on *F*^2^, conventional *R*-factors *R* are based on *F*, with *F* set to zero for negative *F*^2^. The threshold expression of *F*^2^ > σ(*F*^2^) is used only for calculating *R*-factors(gt) *etc*. and is not relevant to the choice of reflections for refinement. *R*-factors based on *F*^2^ are statistically about twice as large as those based on *F*, and *R*- factors based on ALL data will be even larger.

### Fractional atomic coordinates and isotropic or equivalent isotropic displacement parameters (Å^2^)

**Table:**

	*x*	*y*	*z*	*U*_iso_*/*U*_eq_	
W	−0.040028 (18)	0.614346 (12)	0.673068 (7)	0.02272 (7)	
Ti	0.30368 (8)	0.78191 (5)	0.53935 (3)	0.02177 (16)	
Cl	0.55600 (14)	0.83949 (10)	0.58007 (5)	0.0410 (3)	
O1	0.2431 (3)	0.7285 (2)	0.62039 (11)	0.0245 (6)	
O2	−0.1363 (4)	0.6408 (3)	0.52255 (14)	0.0404 (8)	
O3	−0.3832 (4)	0.5174 (3)	0.67496 (16)	0.0509 (9)	
O4	−0.0034 (5)	0.6032 (3)	0.82667 (14)	0.0521 (10)	
O5	0.0917 (5)	0.3753 (3)	0.65428 (18)	0.0569 (10)	
O6	−0.1413 (5)	0.8615 (3)	0.69147 (17)	0.0505 (9)	
C1	0.1989 (4)	0.6800 (3)	0.66995 (16)	0.0207 (8)	
C2	−0.0937 (5)	0.6307 (3)	0.5758 (2)	0.0282 (9)	
C3	−0.2601 (5)	0.5542 (3)	0.67555 (19)	0.0326 (10)	
C4	−0.0115 (5)	0.6062 (3)	0.7719 (2)	0.0331 (10)	
C5	0.0453 (5)	0.4607 (4)	0.66182 (19)	0.0340 (10)	
C6	−0.1093 (5)	0.7727 (4)	0.68423 (19)	0.0332 (10)	
C21	0.3261 (4)	0.6812 (3)	0.72524 (16)	0.0227 (8)	
C22	0.3483 (5)	0.5921 (3)	0.76655 (17)	0.0261 (9)	
H22	0.2780	0.5321	0.7615	0.031*	
C23	0.4726 (5)	0.5901 (4)	0.81522 (19)	0.0337 (10)	
H23	0.4888	0.5281	0.8424	0.040*	
C24	0.5731 (5)	0.6791 (4)	0.82394 (18)	0.0359 (11)	
H24	0.6580	0.6779	0.8572	0.043*	
C25	0.5499 (5)	0.7688 (4)	0.7844 (2)	0.0378 (10)	
H25	0.6173	0.8302	0.7912	0.045*	
C26	0.4280 (5)	0.7699 (3)	0.73471 (17)	0.0300 (9)	
H26	0.4141	0.8314	0.7070	0.036*	
C31	0.0676 (5)	0.8817 (3)	0.5469 (2)	0.0321 (10)	
H31	−0.0155	0.8518	0.5683	0.039*	
C32	0.0882 (5)	0.8697 (3)	0.4812 (2)	0.0337 (10)	
H32	0.0225	0.8290	0.4503	0.040*	
C33	0.2228 (5)	0.9284 (4)	0.4690 (2)	0.0362 (10)	
H33	0.2646	0.9343	0.4285	0.043*	
C34	0.2847 (5)	0.9768 (3)	0.5274 (2)	0.0335 (10)	
H34	0.3745	1.0227	0.5330	0.040*	
C35	0.1924 (5)	0.9463 (3)	0.57560 (19)	0.0319 (10)	
H35	0.2101	0.9654	0.6200	0.038*	
C41	0.2059 (6)	0.6425 (4)	0.4682 (2)	0.0424 (12)	
H41	0.0960	0.6397	0.4555	0.051*	
C42	0.2865 (7)	0.5904 (4)	0.5223 (2)	0.0439 (12)	
H42	0.2405	0.5442	0.5519	0.053*	
C43	0.4452 (7)	0.6184 (3)	0.5251 (2)	0.0458 (13)	
H43	0.5260	0.5968	0.5573	0.055*	
C44	0.4629 (6)	0.6842 (4)	0.4713 (2)	0.0432 (12)	
H44	0.5593	0.7138	0.4604	0.052*	
C45	0.3183 (6)	0.6991 (4)	0.43667 (19)	0.0399 (11)	
H45	0.2983	0.7406	0.3981	0.048*	

### Atomic displacement parameters (Å^2^)

**Table:**

	*U*^11^	*U*^22^	*U*^33^	*U*^12^	*U*^13^	*U*^23^
W	0.02340 (10)	0.02525 (10)	0.01927 (9)	−0.00116 (7)	0.00104 (7)	0.00126 (6)
Ti	0.0253 (4)	0.0243 (4)	0.0157 (3)	0.0017 (3)	0.0022 (3)	0.0023 (3)
Cl	0.0411 (7)	0.0424 (6)	0.0388 (6)	−0.0064 (5)	0.0013 (5)	0.0034 (5)
O1	0.0297 (16)	0.0257 (14)	0.0179 (12)	−0.0016 (12)	0.0008 (11)	0.0041 (11)
O2	0.045 (2)	0.0505 (19)	0.0231 (15)	−0.0082 (16)	−0.0072 (14)	0.0014 (13)
O3	0.0308 (19)	0.066 (2)	0.057 (2)	−0.0159 (18)	0.0105 (16)	−0.0051 (18)
O4	0.060 (2)	0.075 (3)	0.0213 (17)	0.0082 (19)	0.0064 (15)	0.0073 (15)
O5	0.072 (3)	0.037 (2)	0.056 (2)	0.0205 (18)	−0.018 (2)	−0.0063 (16)
O6	0.065 (2)	0.0354 (19)	0.051 (2)	0.0102 (17)	0.0045 (18)	−0.0071 (16)
C1	0.024 (2)	0.0187 (19)	0.0199 (17)	0.0039 (16)	0.0048 (15)	0.0006 (15)
C2	0.026 (2)	0.028 (2)	0.030 (2)	−0.0050 (17)	0.0034 (18)	−0.0016 (17)
C3	0.033 (3)	0.034 (3)	0.030 (2)	0.000 (2)	0.0032 (19)	0.0010 (19)
C4	0.034 (3)	0.039 (3)	0.028 (2)	−0.0027 (19)	0.0086 (19)	0.0048 (18)
C5	0.035 (3)	0.036 (3)	0.028 (2)	0.001 (2)	−0.0089 (18)	0.0017 (19)
C6	0.036 (3)	0.037 (3)	0.026 (2)	0.000 (2)	0.0020 (18)	−0.0027 (19)
C21	0.024 (2)	0.027 (2)	0.0166 (16)	0.0010 (17)	0.0020 (15)	0.0004 (15)
C22	0.028 (2)	0.028 (2)	0.0224 (18)	0.0026 (17)	0.0045 (16)	0.0019 (16)
C23	0.033 (2)	0.044 (3)	0.024 (2)	0.008 (2)	0.0022 (18)	0.0088 (19)
C24	0.027 (2)	0.058 (3)	0.022 (2)	0.007 (2)	−0.0016 (17)	−0.004 (2)
C25	0.031 (2)	0.047 (3)	0.034 (2)	−0.011 (2)	−0.0023 (19)	0.001 (2)
C26	0.032 (2)	0.038 (2)	0.0194 (18)	−0.0063 (19)	0.0004 (17)	0.0061 (17)
C31	0.029 (2)	0.031 (2)	0.038 (2)	0.0088 (19)	0.0072 (19)	0.0127 (18)
C32	0.035 (3)	0.036 (3)	0.027 (2)	0.010 (2)	−0.0108 (19)	0.0062 (17)
C33	0.041 (3)	0.040 (3)	0.029 (2)	0.006 (2)	0.0071 (19)	0.0161 (19)
C34	0.037 (3)	0.021 (2)	0.043 (2)	−0.0007 (19)	0.004 (2)	0.0065 (18)
C35	0.041 (3)	0.024 (2)	0.031 (2)	0.0067 (19)	0.0072 (19)	−0.0009 (17)
C41	0.049 (3)	0.044 (3)	0.035 (2)	−0.003 (2)	0.004 (2)	−0.022 (2)
C42	0.070 (4)	0.027 (2)	0.038 (2)	−0.003 (2)	0.018 (2)	−0.010 (2)
C43	0.063 (4)	0.034 (3)	0.041 (3)	0.021 (2)	0.007 (2)	−0.005 (2)
C44	0.046 (3)	0.049 (3)	0.038 (2)	0.012 (2)	0.018 (2)	−0.007 (2)
C45	0.055 (3)	0.043 (3)	0.023 (2)	0.010 (2)	0.010 (2)	−0.0064 (19)

### Geometric parameters (Å, °)

**Table:**

W—C3	2.028 (5)	C22—H22	0.9500
W—C2	2.038 (4)	C23—C24	1.389 (7)
W—C5	2.043 (5)	C23—H23	0.9500
W—C4	2.047 (4)	C24—C25	1.375 (6)
W—C6	2.051 (5)	C24—H24	0.9500
W—C1	2.204 (4)	C25—C26	1.391 (5)
Ti—O1	1.927 (2)	C25—H25	0.9500
Ti—Cl	2.3446 (14)	C26—H26	0.9500
Ti—C41	2.358 (4)	C31—C32	1.404 (6)
Ti—C32	2.358 (4)	C31—C35	1.411 (6)
Ti—C33	2.374 (4)	C31—H31	0.9500
Ti—C43	2.377 (4)	C32—C33	1.402 (6)
Ti—C42	2.378 (4)	C32—H32	0.9500
Ti—C45	2.379 (4)	C33—C34	1.406 (6)
Ti—C31	2.381 (4)	C33—H33	0.9500
Ti—C35	2.385 (4)	C34—C35	1.389 (5)
Ti—C44	2.385 (4)	C34—H34	0.9500
Ti—C34	2.408 (4)	C35—H35	0.9500
O1—C1	1.280 (4)	C41—C45	1.403 (6)
O2—C2	1.135 (5)	C41—C42	1.410 (7)
O3—C3	1.144 (5)	C41—H41	0.9500
O4—C4	1.134 (5)	C42—C43	1.395 (7)
O5—C5	1.138 (5)	C42—H42	0.9500
O6—C6	1.137 (5)	C43—C44	1.399 (6)
C1—C21	1.499 (5)	C43—H43	0.9500
C21—C22	1.391 (5)	C44—C45	1.377 (7)
C21—C26	1.396 (5)	C44—H44	0.9500
C22—C23	1.390 (6)	C45—H45	0.9500
			
C3—W—C2	86.90 (16)	C21—C1—W	125.7 (2)
C3—W—C5	90.63 (17)	O2—C2—W	174.3 (4)
C2—W—C5	91.35 (16)	O3—C3—W	177.2 (4)
C3—W—C4	88.38 (17)	O4—C4—W	176.6 (4)
C2—W—C4	173.19 (17)	O5—C5—W	178.6 (4)
C5—W—C4	93.62 (16)	O6—C6—W	177.1 (4)
C3—W—C6	93.55 (17)	C22—C21—C26	118.8 (4)
C2—W—C6	88.91 (16)	C22—C21—C1	120.5 (3)
C5—W—C6	175.82 (17)	C26—C21—C1	120.6 (3)
C4—W—C6	86.47 (16)	C23—C22—C21	120.6 (4)
C3—W—C1	179.75 (15)	C23—C22—H22	119.7
C2—W—C1	92.86 (14)	C21—C22—H22	119.7
C5—W—C1	89.45 (15)	C24—C23—C22	119.8 (4)
C4—W—C1	91.85 (15)	C24—C23—H23	120.1
C6—W—C1	86.37 (15)	C22—C23—H23	120.1
O1—Ti—Cl	96.14 (8)	C25—C24—C23	120.1 (4)
O1—Ti—C41	101.07 (14)	C25—C24—H24	120.0
Cl—Ti—C41	134.35 (14)	C23—C24—H24	120.0
O1—Ti—C32	109.71 (14)	C24—C25—C26	120.2 (4)
Cl—Ti—C32	133.57 (12)	C24—C25—H25	119.9
C41—Ti—C32	78.56 (17)	C26—C25—H25	119.9
O1—Ti—C33	135.06 (13)	C25—C26—C21	120.5 (4)
Cl—Ti—C33	101.17 (12)	C25—C26—H26	119.8
C41—Ti—C33	95.76 (17)	C21—C26—H26	119.8
C32—Ti—C33	34.46 (15)	C32—C31—C35	107.7 (4)
O1—Ti—C43	90.49 (14)	C32—C31—Ti	71.9 (2)
Cl—Ti—C43	80.67 (15)	C35—C31—Ti	72.9 (2)
C41—Ti—C43	57.43 (19)	C32—C31—H31	126.1
C32—Ti—C43	134.63 (17)	C35—C31—H31	126.1
C33—Ti—C43	132.96 (16)	Ti—C31—H31	120.8
O1—Ti—C42	77.09 (13)	C33—C32—C31	108.0 (4)
Cl—Ti—C42	113.08 (15)	C33—C32—Ti	73.4 (2)
C41—Ti—C42	34.65 (17)	C31—C32—Ti	73.6 (2)
C32—Ti—C42	109.99 (18)	C33—C32—H32	126.0
C33—Ti—C42	130.25 (17)	C31—C32—H32	126.0
C43—Ti—C42	34.13 (18)	Ti—C32—H32	118.9
O1—Ti—C45	133.08 (14)	C32—C33—C34	107.7 (4)
Cl—Ti—C45	108.73 (13)	C32—C33—Ti	72.1 (2)
C41—Ti—C45	34.45 (16)	C34—C33—Ti	74.2 (2)
C32—Ti—C45	81.11 (16)	C32—C33—H33	126.2
C33—Ti—C45	79.00 (16)	C34—C33—H33	126.2
C43—Ti—C45	56.85 (17)	Ti—C33—H33	119.4
C42—Ti—C45	56.82 (16)	C35—C34—C33	108.5 (4)
O1—Ti—C31	79.09 (13)	C35—C34—Ti	72.3 (2)
Cl—Ti—C31	125.21 (12)	C33—C34—Ti	71.6 (2)
C41—Ti—C31	99.57 (17)	C35—C34—H34	125.8
C32—Ti—C31	34.47 (14)	C33—C34—H34	125.8
C33—Ti—C31	57.05 (14)	Ti—C34—H34	122.1
C43—Ti—C31	152.69 (18)	C34—C35—C31	108.0 (4)
C42—Ti—C31	118.56 (17)	C34—C35—Ti	74.1 (2)
C45—Ti—C31	113.73 (16)	C31—C35—Ti	72.6 (2)
O1—Ti—C35	81.89 (12)	C34—C35—H35	126.0
Cl—Ti—C35	90.79 (11)	C31—C35—H35	126.0
C41—Ti—C35	133.17 (17)	Ti—C35—H35	119.2
C32—Ti—C35	57.28 (15)	C45—C41—C42	107.1 (5)
C33—Ti—C35	56.94 (15)	C45—C41—Ti	73.6 (3)
C43—Ti—C35	167.92 (17)	C42—C41—Ti	73.5 (3)
C42—Ti—C35	149.50 (17)	C45—C41—H41	126.4
C45—Ti—C35	134.76 (15)	C42—C41—H41	126.4
C31—Ti—C35	34.43 (14)	Ti—C41—H41	118.5
O1—Ti—C44	124.66 (14)	C43—C42—C41	108.4 (4)
Cl—Ti—C44	78.77 (13)	C43—C42—Ti	72.9 (2)
C41—Ti—C44	56.67 (18)	C41—C42—Ti	71.9 (3)
C32—Ti—C44	112.91 (16)	C43—C42—H42	125.8
C33—Ti—C44	99.40 (16)	C41—C42—H42	125.8
C43—Ti—C44	34.17 (16)	Ti—C42—H42	121.1
C42—Ti—C44	56.31 (17)	C42—C43—C44	107.1 (5)
C45—Ti—C44	33.62 (16)	C42—C43—Ti	73.0 (3)
C31—Ti—C44	146.91 (16)	C44—C43—Ti	73.2 (2)
C35—Ti—C44	152.05 (16)	C42—C43—H43	126.5
O1—Ti—C34	113.94 (12)	C44—C43—H43	126.5
Cl—Ti—C34	77.75 (11)	Ti—C43—H43	119.3
C41—Ti—C34	129.81 (17)	C45—C44—C43	109.2 (5)
C32—Ti—C34	56.81 (15)	C45—C44—Ti	72.9 (2)
C33—Ti—C34	34.20 (15)	C43—C44—Ti	72.6 (2)
C43—Ti—C34	148.78 (17)	C45—C44—H44	125.4
C42—Ti—C34	164.43 (16)	C43—C44—H44	125.4
C45—Ti—C34	109.94 (15)	Ti—C44—H44	120.7
C31—Ti—C34	56.44 (14)	C44—C45—C41	108.1 (4)
C35—Ti—C34	33.69 (13)	C44—C45—Ti	73.4 (2)
C44—Ti—C34	118.36 (16)	C41—C45—Ti	72.0 (2)
C1—O1—Ti	171.7 (2)	C44—C45—H45	125.9
O1—C1—C21	111.2 (3)	C41—C45—H45	125.9
O1—C1—W	123.0 (3)	Ti—C45—H45	120.4

## References

[bb1] Atwood, J. L. & Barbour, L. J. (2003). *Cryst. Growth Des.* **3**, 3–8.

[bb2] Balzer, B. L., Cazanoue, M., Sabat, M. & Finn, M. G. (1992). *Organometallics*, **11**, 1759–1761.

[bb3] Barbour, L. J. (2001). *J. Supramol. Chem.* **1**, 189–191.

[bb4] Barluenga, J. & Fañanás, F. J. (2000). *Tetrahedron*, **56**, 4597–4628.

[bb5] Esterhuysen, C., Nel, I. B. J. & Cronje, S. (2008). *Acta Cryst.* E**64**, m1150. 10.1107/S1600536808025245 PMC2960508 21201603

[bb6] Luruli, N., Grumel, V., Brüll, R., Du Toit, A., Pasch, H., Van Reenen, A. J. & Raubenheimer, H. G. (2004). *J. Polym. Sci* A**1**, 5121–5133.

[bb7] Luruli, N., Heinz, L. C., Grumel, V., Brüll, R., Pasch, H. & Raubenheimer, H. G. (2006). *Polymer*, **47**, 56–66.

[bb8] Nonius (1998). *COLLECT* Nonius BV, Delft, The Netherlands.

[bb9] Orpen, A. G., Brammer, L., Allen, F. H., Kennard, O., Watson, D. G. & Taylor, R. (1989). *J. Chem. Soc. Dalton Trans.* pp. S1–83.

[bb10] Otwinowski, Z. & Minor, W. (1997). *Methods in Enzymology*, Vol. 276, *Macromolecular Crystallography*, Part A, edited by C. W. Carter Jr & R. M. Sweet, pp. 307–326. New York: Academic Press.

[bb11] Sheldrick, G. M. (2008). *Acta Cryst.* A**64**, 112–122. 10.1107/S0108767307043930 18156677

[bb12] Sinn, H., Kaminsky, W., Vollmer, H. J. & Woldt, R. (1980). * Angew. Chem. Int. Ed. Engl* **19**, 390–392.

[bb13] Westrip, S. P. (2008). *publCIF* In preparation.

